# Changes in the gut microbiota of patients with sarcopenia based on 16S rRNA gene sequencing: a systematic review and meta-analysis

**DOI:** 10.3389/fnut.2024.1429242

**Published:** 2024-06-28

**Authors:** Qi Song, Youkang Zhu, Xiao Liu, Hai Liu, Xinyi Zhao, Liyun Xue, Shaoying Yang, Yujia Wang, Xifang Liu

**Affiliations:** ^1^Honghui Hospital, Xi’an Jiaotong University, Xi’an, China; ^2^Xi’an Physical Education University, Xi’an, China; ^3^Xi’an Jiaotong University, Xi’an, China

**Keywords:** gut microbiota, sarcopenia, 16S rRNA, meta-analysis, nutrition

## Abstract

**Introduction:**

Sarcopenia, an age-related disease, has become a major public health concern, threatening muscle health and daily functioning in older adults around the world. Changes in the gut microbiota can affect skeletal muscle metabolism, but the exact association is unclear. The richness of gut microbiota refers to the number of different species in a sample, while diversity not only considers the number of species but also the evenness of their abundances. Alpha diversity is a comprehensive metric that measures both the number of different species (richness) and the evenness of their abundances, thereby providing a thorough understanding of the species composition and structure of a community.

**Methods:**

This meta-analysis explored the differences in intestinal microbiota diversity and richness between populations with sarcopenia and non-sarcopenia based on 16 s rRNA gene sequencing and identified new targets for the prevention and treatment of sarcopenia. PubMed, Embase, Web of Science, and Google Scholar databases were searched for cross-sectional studies on the differences in gut microbiota between sarcopenia and non-sarcopenia published from 1995 to September 2023 scale and funnel plot analysis assessed the risk of bias, and performed a meta-analysis with State v.15. 1.

**Results:**

A total of 17 randomized controlled studies were included, involving 4,307 participants aged 43 to 87 years. The alpha diversity of intestinal flora in the sarcopenia group was significantly reduced compared to the non-sarcopenia group: At the richness level, the proportion of Actinobacteria and Fusobacteria decreased, although there was no significant change in other phyla. At the genus level, the abundance of f-Ruminococcaceae; g-*Faecalibacterium*, g-*Prevotella*, *Lachnoclostridium*, and other genera decreased, whereas the abundance of g-*Bacteroides*, *Parabacteroides*, and *Shigella* increased.

**Discussion:**

This study showed that the richness of the gut microbiota decreased with age in patients with sarcopenia. Furthermore, the relative abundance of different microbiota changed related to age, comorbidity, participation in protein metabolism, and other factors. This study provides new ideas for targeting the gut microbiota for the prevention and treatment of sarcopenia.

**Systematic Review Registration:**

https://www.crd.york.ac.uk/PROSPERO/display_record.php?RecordID=475887, CRD475887.

## 1 Introduction

Sarcopenia is a degenerative disease with age-related loss of skeletal muscle mass and strength, which is implicated in the occurrence of adverse outcomes including falls, fractures, physical disability, and death. Loss of muscle mass over the course of life development starts at age 40 and decreases by approximately 8% every 10 years, accelerating to 15% after age 70 and lasting until death (1). Sarcopenia has a high prevalence: 5–13% in individuals aged 60–70 and 11–50% in those aged 80 and older (2). Multiple in-depth studies have found that assessing muscle strength is a stronger link to sarcopenia than muscle mass. As a result, international sarcopenia research organizations such as the European Sarcopenia Working Group (EWGSOP2) and the Asian Sarcopenia Working Group (AWGS) introduced a new definition of sarcopenia diagnosis in 2019, focusing more on muscle strength (3, 4). The diagnosis of sarcopenia was established using a combination of criteria, including low muscle mass, diminished muscle strength, and impaired physical performance. Specifically, muscle mass was evaluated using techniques such as dual-energy X-ray absorptiometry (DXA) or bioelectrical impedance analysis (BIA). The thresholds for low muscle mass were defined as <7.0 kg/m^2^ for men and < 5.5 kg/m^2^ for women. Muscle strength was typically assessed through handgrip strength tests, with cut-off values of <27 kg for men and < 16 kg for women. Physical performance was gaged by measuring gait speed, with a threshold of <0.8 m/s, or by using the Short Physical Performance Battery (SPPB). These criteria align with the recommendations of the European Working Group on Sarcopenia in Older People (EWGSOP) and other relevant guidelines (4, 5). According to the EWGSOP guidelines, sarcopenia is diagnosed based on the presence of both low muscle mass and low muscle function (strength or performance). The stages of sarcopenia are further classified as presarcopenia (low muscle mass alone), sarcopenia (low muscle mass plus low muscle strength or performance), and severe sarcopenia (low muscle mass, low muscle strength, and low physical performance). However, due to the complex pathophysiology of sarcopenia involving multiple related pathways and limited understanding, there is currently a lack of single effective targeted therapy drugs in clinical treatment.

In recent years, the gut microbiota has received significant attention as a regulator of inflammatory response and anabolic balance. The human gut microbiota defines the microorganisms that live in the human gastrointestinal tract and co-exist with hosts. The microbiota is made up of about 10 to 100 trillion microorganisms. As the largest and most complex microbial system of human body, intestinal microbes and their metabolites play a vital role in the immune and endocrine functions of the gut, energy homeostasis, and health maintenance of the body. The number and composition of the microbiome changes with age. For example, the core symbiotic bacterial species Bacteroides, Bifidobacteriales and others decrease, while the opportunistic microorganisms such as Fusobacterium and *Escherichia coli* increase (6). Recent studies have found that the gut microbiota and its metabolites can act on muscles, so the theory of “gut-muscle axis” has been proposed (7). Previous studies have found that intestinal microbiota dysregulation is associated with loss of skeletal muscle mass and function. Yue et al. showed that the diversity and richness of the intestinal microbiota in patients with sarcopenia were lower than the control group. Specifically, the reduction in the ratio of Prevotella to Bacteroidetes (P/B), as well as the decreased abundance of Coprococcus and Lachnospiraceae, were significant indicators. Prevotella and Bacteroidetes are involved in the fermentation of dietary fibers and production of short-chain fatty acids (SCFAs), which are crucial for maintaining muscle mass and function. A lower P/B ratio suggests a reduced capacity for SCFA production, which can negatively impact muscle health. Similarly, Coprococcus and Lachnospiraceae are SCFA-producing bacteria, and their reduction is associated with decreased SCFA levels, leading to muscle atrophy and weakness. Therefore, monitoring these specific bacterial markers can provide early indications of sarcopenia, allowing for timely interventions (8). Lee et al., in a study of older adults in a community setting, reported significant decrease in abundances of Prevotella and fecal Prevotella (*p* = 0.021 and *p* = 0.018) and significant increase in abundances of Paracytobacter (*p* = 0.010) in patients with sarcopenia (9). Furthermore, age-related microbial dysbiosis is associated with increased permeability of the intestinal mucosal barrier function (10), which promotes the release of toxins produced by microbials into the circulation and leads to inflammation (11). The ubiquitin proteasome system (UPS) plays a critical role in the degradation of damaged or misfolded proteins and the regulation of various cellular processes, including muscle protein turnover. In skeletal muscle, the UPS is responsible for the breakdown of myofibrillar proteins, which are essential components of muscle fibers. Dysregulation of the UPS can lead to excessive protein degradation, resulting in muscle atrophy and a decrease in skeletal muscle mass. Studies have shown that increased activity of the UPS is associated with muscle wasting conditions, such as sarcopenia (12, 13). The gut microbiota can influence the UPS through the production of metabolites, such as short-chain fatty acids (SCFAs), which are released in fermentation processes associated with fiber digestion and have been shown to modulate inflammation and protein synthesis pathways (14, 15). Therefore, alterations in the gut microbiota that reduce SCFA production can indirectly enhance UPS activity, contributing to the loss of muscle mass observed in sarcopenia.

Over the past few years, research has increasingly focused on the composition of the gut microbiota in relation to sarcopenia. Several orders, taxa, families, and genera have been identified as key indicators in these studies. For instance, the order Clostridiales, which includes many beneficial gut bacteria, has been frequently examined (16). At the phylum level, Firmicutes and Bacteroidetes are often scrutinized, with studies indicating a decrease in their abundance in individuals with sarcopenia (17). At the family level, Lachnospiraceae and Ruminococcaceae are noted for their roles in gut health and metabolism, with lower levels observed in sarcopenic individual (18). Additionally, the family Coriobacteriaceae, including the genus *Collinsella*, has been noted for its potential pathogenic role and altered abundance in sarcopenic patients (19). At the genus level, *Prevotella* and *Coprococcus* have been identified as significant markers, with research showing their reduced abundance in sarcopenic populations (16, 17). These taxa are believed to influence inflammation, energy metabolism, and muscle health, making them relevant indicators for sarcopenia research.

16S ribosomal RNA (16 s rRNA), as a gene widely present in all bacterial genomes, can be used as a sequencing target for the proliferation and development of microbial systems, as well as a bridge to link the transgenic effects and disease characteristics. The 16 s bacterial rRNA sequence can detect the structural characteristics of intestinal microbes in feces, which facilitates understanding of changes in the intestinal microbiota in different populations (20). Using the 16S rRNA sequencing method, Kenta Yamamoto tested the intestinal microbial diversity and richness of 69 patients living in the community and found that the F/B value of patients with sarcopenia was reduced, and at the genus level, *Coprobacillus*, *Catenibacterium*, and Clostridium were also lower, whereas the level of *Bacteroides* was higher (20). This may be related to the effect of gut microbiome on muscle synthesis and breakdown. The decrease in *Coprobacillus*, *Catenibacterium*, and Clostridium, which are known to produce short-chain fatty acids (SCFAs) through the fermentation of dietary fibers, can lead to a reduction in SCFA levels. SCFAs play a crucial role in regulating inflammation and promoting muscle protein synthesis. On the other hand, the increase in Bacteroides, which is associated with the breakdown of complex carbohydrates and the production of other metabolites, may result in an imbalance in the gut microbiota that favors muscle protein breakdown over synthesis. This imbalance can exacerbate the muscle atrophy and weakness observed in sarcopenia (15, 21, 22). However, the results of 16S rRNA sequencing are inconsistent and lack of systematic summary, which cannot provide a clear reference value for the prevention and clinical treatment of sarcopenia. Therefore, this study aimed to perform a systematic review and meta-analysis of Alpha diversity and richness of gut microbiota (GM) in the population of sarcopenia (SAR) and non-sarcopenia (N-SAR), in order to clarify the role of GM and its metabolites in the pathogenesis of sarcopenia, providing new ideas and methods for the treatment of sarcopenia.

## 2 Materials and methods

### 2.1 Search strategies

This study was prospectively registered with PROSPERO following the PRISMA guidelines (CRD475887). A comprehensive search was conducted in PubMed, Embase, Web of Science, and Google Scholar using keywords and free words based on the PICOS principle. Additionally, a literature search and manual search were performed. The search was conducted from 1995 until 5 September 2023, without any language or date restrictions. The specific search strategy is depicted in the Table 1.

**Table 1:** Search strategies.

Search	Query	Results
#4	Search:(“sarcopenia”[MeSH Terms] OR “Sarcopenias”[Title/Abstract]) AND (“rna, ribosomal, 16 s”[MeSH Terms] OR “16 s rrna”[Title/Abstract] OR “rrna 16 s”[Title/Abstract] OR “16 s ribosomal rna”[Title/Abstract] OR “rna 16 s ribosomal”[Title/Abstract] OR “ribosomal rna 16 s”[Title/Abstract]) AND (“gastrointestinal microbiome”[MeSH Terms] OR “gastrointestinal microbiomes”[Title/Abstract] OR “microbiome gastrointestinal”[Title/Abstract] OR “gut microbiome”[Title/Abstract] OR “gut microbiomes”[Title/Abstract] OR “microbiome gut”[Title/Abstract] OR “gut microflora”[Title/Abstract] OR “microflora gut”[Title/Abstract] OR “gut microbiota”[Title/Abstract] OR “gut microbiotas”[Title/Abstract] OR “microbiota gut”[Title/Abstract] OR “gastrointestinal flora”[Title/Abstract] OR “flora gastrointestinal”[Title/Abstract] OR “gut flora”[Title/Abstract] OR “flora gut”[Title/Abstract] OR “gastrointestinal microbiota”[Title/Abstract] OR “gastrointestinal microbiotas”[Title/Abstract] OR “microbiota gastrointestinal”[Title/Abstract] OR “gastrointestinal microbial community”[Title/Abstract] OR “gastrointestinal microbial communities”[Title/Abstract] OR ((“Microbiota”[MeSH Terms] OR “Microbiota”[All Fields] OR (“Microbial”[All Fields] AND “Community”[All Fields]) OR “microbial community”[All Fields]) AND “Gastrointestinal”[Title/Abstract]) OR “gastrointestinal microflora”[Title/Abstract] OR “microflora gastrointestinal”[Title/Abstract] OR “gastric microbiome”[Title/Abstract] OR “gastric microbiomes”[Title/Abstract] OR “microbiome gastric”[Title/Abstract] OR “intestinal microbiome”[Title/Abstract] OR “intestinal microbiomes”[Title/Abstract] OR “microbiome intestinal”[Title/Abstract] OR “intestinal microbiota”[Title/Abstract] OR “intestinal microbiotas”[Title/Abstract] OR “microbiota intestinal”[Title/Abstract] OR “intestinal microflora”[Title/Abstract] OR “microflora intestinal”[Title/Abstract] OR “intestinal flora”[Title/Abstract] OR “flora intestinal”[Title/Abstract] OR “enteric bacteria”[Title/Abstract] OR “bacteria enteric”[Title/Abstract])	12
#3	Search: ((((((((((((((((((((((((((((((((((((((Gastrointestinal Microbiome[MeSH Terms])) OR (Gastrointestinal Microbiomes[Title/Abstract])) OR (Microbiome, Gastrointestinal[Title/Abstract])) OR (Gut Microbiome[Title/Abstract])) OR (Gut Microbiomes[Title/Abstract])) OR (Microbiome, Gut[Title/Abstract])) OR (Gut Microflora[Title/Abstract])) OR (Microflora, Gut[Title/Abstract])) OR (Gut Microbiota[Title/Abstract])) OR (Gut Microbiotas[Title/Abstract])) OR (Microbiota, Gut[Title/Abstract])) OR (Gastrointestinal Flora[Title/Abstract])) OR (Flora, Gastrointestinal[Title/Abstract])) OR (Gut Flora[Title/Abstract])) OR (Flora, Gut[Title/Abstract])) OR (Gastrointestinal Microbiota[Title/Abstract])) OR (Gastrointestinal Microbiotas[Title/Abstract])) OR (Microbiota, Gastrointestinal[Title/Abstract])) OR (Gastrointestinal Microbial Community[Title/Abstract])) OR (Gastrointestinal Microbial Communities[Title/Abstract])) OR (Microbial Community, Gastrointestinal[Title/Abstract])) OR (Gastrointestinal Microflora[Title/Abstract])) OR (Microflora, Gastrointestinal[Title/Abstract])) OR (Gastric Microbiome[Title/Abstract])) OR (Gastric Microbiomes[Title/Abstract])) OR (Microbiome, Gastric[Title/Abstract])) OR (Intestinal Microbiome[Title/Abstract])) OR (Intestinal Microbiomes[Title/Abstract])) OR (Microbiome, Intestinal[Title/Abstract])) OR (Intestinal Microbiota[Title/Abstract])) OR (Intestinal Microbiotas[Title/Abstract])) OR (Microbiota, Intestinal[Title/Abstract])) OR (Intestinal Microflora[Title/Abstract])) OR (Microflora, Intestinal[Title/Abstract])) OR (Intestinal Flora[Title/Abstract])) OR (Flora, Intestinal[Title/Abstract])) OR (Enteric Bacteria[Title/Abstract])) OR (Bacteria, Enteric[Title/Abstract])	95,471
#2	Search: (((((RNA, Ribosomal, 16S[MeSH Terms]) OR (16S rRNA[Title/Abstract])) OR (rRNA, 16S[Title/Abstract])) OR (16S Ribosomal RNA[Title/Abstract])) OR (RNA, 16S Ribosomal[Title/Abstract])) OR (Ribosomal RNA, 16S[Title/Abstract])	96,192
#1	Search: (Sarcopenia[MeSH Terms]) OR (Sarcopenias[Title/Abstract])	10,433
#4	#1 AND #2 AND #3	32
#3	‘gastrointestinal microbiome’:ab,ti OR ‘gastrointestinal microbiomes’:ab,ti OR ‘microbiome, gastrointestinal’:ab,ti OR ‘gut microbiome’:ab,ti OR ‘gut microbiomes’:ab,ti OR ‘microbiome, gut’:ab,ti OR ‘gut microflora’:ab,ti OR ‘microflora, gut’:ab,ti OR ‘gut microbiota’:ab,ti OR ‘gut microbiotas’:ab,ti OR ‘microbiota, gut’:ab,ti OR ‘gastrointestinal flora’:ab,ti OR ‘flora, gastrointestinal’:ab,ti OR ‘gut flora’:ab,ti OR ‘flora, gut’:ab,ti OR ‘gastrointestinal microbiota’:ab,ti OR ‘gastrointestinal microbiotas’:ab,ti OR ‘microbiota, gastrointestinal’:ab,ti OR ‘gastrointestinal microbial community’:ab,ti OR ‘gastrointestinal microbial communities’:ab,ti OR ‘microbial community, gastrointestinal’:ab,ti OR ‘gastrointestinal microflora’:ab,ti OR ‘microflora, gastrointestinal’:ab,ti OR ‘gastric microbiome’:ab,ti OR ‘gastric microbiomes’:ab,ti OR ‘microbiome, gastric’:ab,ti OR ‘intestinal microbiome’:ab,ti OR ‘intestinal microbiomes’:ab,ti OR ‘microbiome, intestinal’:ab,ti OR ‘intestinal microbiota’:ab,ti OR ‘intestinal microbiotas’:ab,ti OR ‘microbiota, intestinal’:ab,ti OR ‘intestinal microflora’:ab,ti OR ‘microflora, intestinal’:ab,ti OR ‘intestinal flora’:ab,ti OR ‘flora, intestinal’:ab,ti OR ‘enteric bacteria’:ab,ti OR ‘bacteria, enteric’:ab,ti	100,179
#2	rna, ribosomal, 16 s’:ab,ti OR ‘16 s rrna’:ab,ti OR ‘rrna, 16 s’:ab,ti OR ‘16 s ribosomal rna’:ab,ti OR ‘rna, 16 s ribosomal’:ab,ti OR ‘ribosomal rna, 16 s’:ab,ti	83,243
#1	sarcopenia:ab,ti OR sarcopenias:ab,ti	23,983
#4	#1AND #2 AND #3	27
#3	((((((((((((((((((((((((((((((((((((((TS = (Gastrointestinal Microbiome)) OR TS = (Gastrointestinal Microbiomes)) OR TS = (Microbiome, Gastrointestinal)) OR TS = (Gut Microbiome)) OR TS = (Gut Microbiomes)) OR TS = (Microbiome, Gut)) OR TS = (Gut Microflora)) OR TS = (Microflora, Gut)) OR TS = (Gut Microbiota)) OR TS = (Gut Microbiotas)) OR TS = (Microbiota, Gut)) OR TS = (Gastrointestinal Flora)) OR TS = (Flora, Gastrointestinal)) OR TS = (Gut Flora)) OR TS = (Flora, Gut)) OR TS = (Gastrointestinal Microbiota)) OR TS = (Gastrointestinal Microbiotas)) OR TS = (Gastrointestinal Microbiotas)) OR TS = (Microbiota, Gastrointestinal)) OR TS = (Gastrointestinal Microbial Community)) OR TS = (Gastrointestinal Microbial Communities)) OR TS = (Microbial Community, Gastrointestinal)) OR TS = (Gastrointestinal Microflora)) OR TS = (Microflora, Gastrointestinal)) OR TS = (Gastric Microbiome)) OR TS = (Gastric Microbiomes)) OR TS = (Microbiome, Gastric)) OR TS = (Intestinal Microbiome)) OR TS = (Intestinal Microbiomes)) OR TS = (Microbiome, Intestinal)) OR TS = (Intestinal Microbiota)) OR TS = (Intestinal Microbiotas)) OR TS = (Microbiota, Intestinal)) OR TS = (Intestinal Microflora)) OR TS = (Microflora, Intestinal)) OR TS = (Intestinal Flora)) OR TS = (Flora, Intestinal)) OR TS = (Enteric Bacteria)) OR TS = (Bacteria, Enteric)	141,030
#2	(((((TS = (RNA, Ribosomal, 16S)) OR TS = (16S rRNA)) OR TS = (rRNA, 16S)) OR TS = (16S Ribosomal RNA)) OR TS = (RNA, 16S Ribosomal)) OR TS = (Ribosomal RNA, 16S)	101,766
#1	(TS = (Sarcopenia)) OR TS = (Sarcopenias)	25,361

Embase (from inception to 5 September 2023). Web of Science (from inception to 5 September 2023). PubMed (from inception to 5 September 2023).

### 2.2 Search strategy and selection criteria

Inclusion criteria were: (i) cross-sectional clinical study; (ii) studies on the interaction between intestinal microbiota and sarcopenia; (iii) studies that distinguish between sarcopenia and non-sarcopenia based on definitive guidelines, (iv) the use of alpha diversity and/or gut flora richness as outcome indicators.

Exclusion criteria were listed as following: (i) reviews and meta-analysis articles; (ii) meetings and summary publications; (iii) studies that do not involve the interaction between intestinal microbiota and sarcopenia; (iv) studies in animal models or *in vitro* studies; (v) randomized controlled trials, cohort studies, and other non-cross-sectional clinical studies; and (vi) articles that lacked the corresponding data or cannot be extracted from the images.

### 2.3 Study selection

Studies retrieved from different databases were imported into the EndNote X9 bibliography management software for consolidation and deduplication. Two researchers independently read the title and abstract of the literature. According to the inclusion and exclusion criteria, unrelated literature such as nonrandomized controlled trials, cross-sectional studies, meeting minutes, reviews and meta-analyses were excluded. If inclusion in the study could not be determined by reading the title and abstract, it was screened by reading the full text. After the screening, the two researchers cross-checked and compared the final remaining literature. If the same literature was included directly or if there was any disagreement, the inclusion literature was discussed and negotiated.

### 2.4 Data extraction

Data was also compared after extraction by two researchers separately to avoid bias. The literature using gut microbiota diversity assessment employed alpha diversity, which is based on the total number of species, the relative abundance of species, or a combination of both dimensions. At the same time, the phylum and genus richness and relative abundance of bacteria were extracted for analysis. The principle of data extraction was to collect the sample size (n), mean (mean), and standard deviation (SD) of each outcome indicator variable in the sarcopenia and non-sarcopenia populations for analysis. If the mean and standard deviation were not available in the original study, the median and quartile were used to extract and transform. When complete data was not available in the original literature and needed to be extracted from images, the software Engauge Digitizer was utilized to convert these images into specific data by tracing point values. Engauge Digitizer, an open-source software, facilitates the extraction of data points from images of graphs and plots. One of its significant advantages is its capability to process various image formats and its user-friendly interface, which aids in the precise digitization of data. However, it does have some limitations, such as possible inaccuracies when extracting data from low-resolution images and the occasional need for manual adjustments. Despite these drawbacks, Engauge Digitizer remains a valuable tool for researchers requiring the digitization of graphical data (23).

### 2.5 Evaluation of study quality

The included studies were assessed using the Newcastle-Ottawa scale (NOS), which evaluates selection, comparability, and exposure with a maximum score of 9. The final score < 6 was defined as low quality literature, and the final score ≥ 6 was defined as high quality literature. Differences between the two researchers during the evaluation process were resolved through consultation with a third reviewer.

### 2.6 Statistical analyses

In this study, the software State v.15.1 was used for meta-analysis. We used pin-two meta-analyses to compare groups, each for a selected outcome (richness index and diversity index). In these meta-analyses, the effect size was calculated from the standard deviation between the SAR and N-SAR groups, and a forest map was drawn using alpha = 0.05 as the test criterion to describe the statistical results. Heterogeneity between results was assessed statistically using I^2^: If heterogeneity was low (I^2^ < 50%), a fixed-effect model was used for analysis. If heterogeneity is high (I^2^ > 50%), a random effects model was used; the sensitivity was analyzed and the source of heterogeneity was discussed. Publication bias was assessed by funnel plot, the Egger test, and Begg test. If the funnel plot was asymmetrical, it indicated publication bias, which could be quantified by the Egger and Begg tests. When Egger’s or Begg’s test values are >0.05, we consider that there is no publication bias.

## 3 Results

### 3.1 Study selection

**Figure 1:**
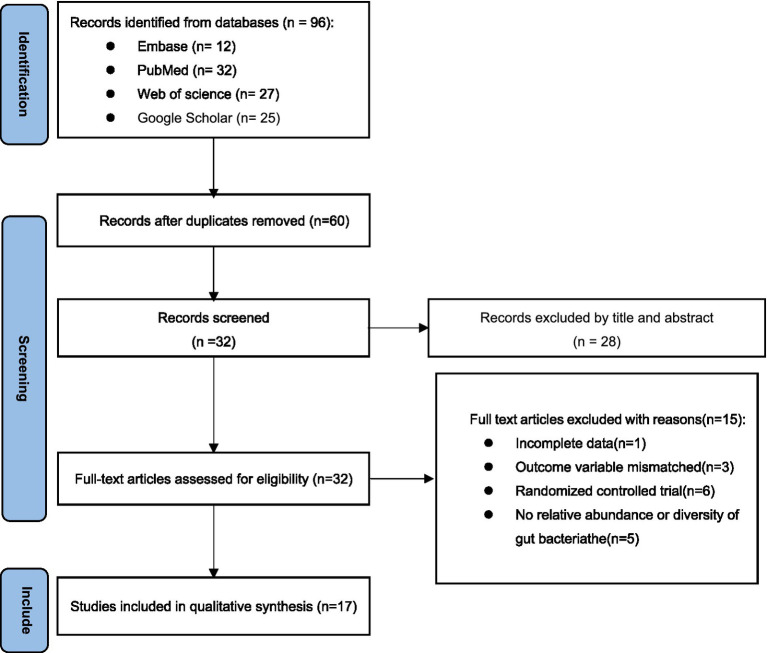
Preferred reporting items for systematic reviews and meta-analysis (PRISMA) flow diagram.

The screening process involved retrieving a total of 96 articles from four electronic databases: PubMed (*n* = 12), Embase (*n* = 32), Web of Science (*n* = 27), and Google Scholar (*n* = 25). After removing duplicates, there were 60 articles remained. The titles and abstracts of these articles were then reviewed, leading to the exclusion of 28 articles. The remaining 32 articles were read in full text, resulting in the exclusion of 15 articles. One article was also excluded due to incomplete data during the data extraction process. Three studies were excluded due to mismatching outcome variables. Six articles were excluded because they were randomized controlled trials rather than cross-sectional studies. Five articles were excluded because they did not provide sufficient alpha diversity or relative abundance. Ultimately, 17 cross-sectional clinical research articles were included (8, 9, 17, 20, 24–36) (Figure 1).

### 3.2 Characteristics of the studies

A total of 17 cross-sectional studies related to the gut microbiota of patients with sarcopenia were published between 2019 and 2023, involving both men and women, and a total of 4,307 stool samples were generated for biome analysis. The amplified regions of the 16sRNA gene varied from study to study. Eleven studies were in V3 to V4, one in V4, and three studies did not report specific sequencing regions. Two other studies were sequenced using metagenomics. In the definition of less muscle disease, different standards of different research applications were used, see Table 2.

**Table 2:** Study and participant characteristics.

Country	First Author, Year	Participants	N	Age(Mean ± SD)	Gender	BMI(Mean ± SD)	SMI(Mean ± SD)	Frailty criteria	Cut-off	Methods of IM assessmalet
Italy	Picca et al. (31)	Community dwellers	35	S:75.5 ± 3.9 NS:73.9 ± 3.2	Both	S:32.14 ± 6.02 NS:26.27 ± 2.55	ND	aLM/BMI aLM	S:aLM/BMI <0.789 in male and < 0.512 in female or aLM <19.75 in male and < 15.02 in female	16 s rRNA sequencing of V3-V4,for alpha diversity and relative abundance
Japan	Yamamoto et al. (20)	Hospitalized	69	S:68 (62.0–73.3) NS:66 (57.5–71.5)	Both	ND	S:37.2 (35.6–39.7) NS:47.1 (43.9–50.7)	Japan Society of Hepatology guidelines for sarcopenia in liver disease (1st edition)	S:42 cm2/m2 in male (AUC, 0.83; sensitivity, 89%; specificity,57%) 38 cm2/m2 in female (AUC, 0.85; sensitivity, 95%; specificity, 96%)	16 s rRNA sequencing of V3-V4,for alpha diversity and relative abundance
Italy	Ticinesi et al. (32)	Community dwellers	17	S:77 (75.5–86) NS:71.5 (70–75)	Both	S:24.3 (20.9–26.7) NS:27.4(24.5–29.1)	S:6.40 (6.33–6.47) NS:7.24 (7.04–9.44)	SPPB score and SMI by BIA considering The First European Consensus on Sarcopenia	S:SPPB score between 3/12 and 9/12 and SMI < 8.87 kg/m2 in male and < 6.42 kg/m2 in females	Shotgun metagenomics sequencing for alpha diversity and relative abundance
China	Han et al. (26)	Hospitalized	88	S:72.3 ± 5.4 NS:70.0 ± 4.2	Both	S:19.7 ± 1.7 NS:22.5 ± 2.2	S:5.65 ± 0.64 NS:6.89 ± 1.02	IWGS	S:SMI <7.23 kg/m2 in male and < 5.67 kg/m2 in female	16 s rRNA sequencing of V3-V4,for alpha diversity and relative abundance
United Kingdom	Cox et al. (24)	Community dwellers	104	S:67.97 (5.67) NS:67.64 (5.43)	Female	S:5.82 (0.69) NS:6.02 (0.80)	ND	EWGSOP2	SMI < 0.6 kg/m2 in female participants and low muscle strength (chair stand time > 15 s for five rises)	16 s rRNA sequencing of V4,for alpha diversity and relative abundance
Australia	Davis et al. (25)	Community dwellers	485	Both:64.4 (13.6)	male	ND	Both:8.5 (0.9)	ND	Low SMI <7.0 kg/m2	16 s rRNA sequencing of V3-V4,for alpha diversity and relative abundance
China	Lee et al. (28)	Hospitalized	50	S:62.7 (54.3–66.5) NS:58.8 (50.9–63.6)	Both	S:22.4 (21.1–23.6) NS:27.5 (25.1–29.4)	S:15.58 ± 1.75 NS:18.75 ± 1.77	AWGS 2019	S:handgrip strength <28 kg in male and < 18 kg in female	16 s rRNA sequencing of V3-V4,for alpha diversity and relative abundance
Japan	Ishida et al. (27)	Nursing Home	23	Both:86.4 ± 7.5	Both	low microbiota:18.5(2.6) hight microbiota: 20.8(2.3)	ND	HS, 5CST, and Calf circumference	Low microbiota:5CST:10.2 ± 3.3 s; Others ND hight microbiota: 5CST:16.3 ± 8.6 s; Others ND	16 s rRNA sequencing
China	Kang et al. (17)	Hospitalized	71	S:76.45 ± 8.58 NS:68.38 ± 5.79	Both	S:20.67 ± 3.27 NS:23.66 ± 2.49	S:6.97 ± 1.38 NS:7.84 ± 0.78	HS,5CST and BIA	Grip strength <28 kg (male) or < 18 kg (female); OR (2) five-time chair stand test ≥12 s. Sarcopenia was defined as muscle mass < 7.0 kg/m2 in male or < 5.7 kg/m2 in female by BIA, in addition to meeting criteria for possible sarcopenia above	16 s rRNA sequencing of V3-V4,for alpha diversity and relative abundance
Korea	Lee et al. (28)	Hospitalized	60	S:66.5(4.6) NS: 64.8 (3.4)	Both	S:23 (3.4) NS:26.4 (3.5)	S:23.0 ± 3.4 kg/m2 NS:26.4 ± 3.5 kg/m2	HS,6MWT and Total limb lean mass/(height)2 (kg/m2)	HGS <28 kg in male, < 18 kg in female; 6-m walk test speed <1 m/s;<7.0 kg/m2 in male and < 5.7 kg/m2 in female	16 s rRNA sequencing of V3-V4,for alpha diversity and relative abundance
Korea	Park et al. (29)	Community dwellers	1,052	S:46.03 ± 8.33 NS:43.66 ± 7.79	Both	S:23.88 ± 2.08 NS:21.95 ± 2.37	ND	SMI (%)by BIA = skeletal muscle mass (kg)/ weight (kg) × 100	<31.0 in male or < 21.0 in female by bioelectrical impedance analysis	16 s rRNA sequencing of V3-V4,for alpha diversity and relative abundance
China	Wu et al. (13)	Hospitalized	192	S:77(65–95) NS:70(65–84)	Both	S:22.8 ± 3.17 N:23.52 ± 30.39	ND	EWGSOP2	S: (1) Low muscle strength <27 kg, (2) low appendicular muscle mass < 20 kg, (3) low physical performance measured with Short Physical Performance Battery eight points	16 s rRNA sequencing of V3-V4,for alpha diversity and relative abundance
China	Peng et al. (30)	Hospitalized	44	S:75.14 ± 8.18 NS:67.67 ± 9.76	Both	S:20.27 ± 3.75 NS:23.52 ± 3.12	S:5.73 ± 0.77 NS:7.14 ± 1.02	AWGS 2019	< 7.0 kg/m2 in male or < 5.7 kg/m2 in female by bioelectrical impedance analysis	16 s rRNA sequencing of V3-V4,for alpha diversity and relative abundance
China	Wang et al. (37)	Community dwellers	1,417	S:72.2(8.5) NS: 62.3 (8.5)	Both	S:21.4 (2.5) NS:24.2 (3.4)	ND	AWGS 2019	①grip strength was low (<28 kg for male and < 18 kg for female); ②SPPB score of ≤9,;③5-time chair stand test of ≥12 s; ④gait speed of <1.0 m/s; ⑤skeletal muscle mass (<7.0 kg/m2 in male and < 5.7 kg/m2 in female)	Shotgun metagenomic sequencing for alpha diversity and relative abundance
China(#)	Yan et al. (35)	Community dwellers	276	S:75.26(7.14) NS: 70.26 (6.03)	Both	S:23.6 (3.07) NS:25.88 (3.67)	S:5.39 ± 0.33 NS:6.62 ± 0.61【ASMI】	AWGS 2019	In female: A person who has low muscle mass (ASMI <5.7 kg/m2) and poor muscle strength (Grip strength <18 kg) or physical performance (6 m gait speed <1.0 m/s)	16 s rRNA sequencing for alpha diversity and relative abundance
China	Zhou et al. (18)	Hospitalized	60	S: 49.9(12.6) NS: 45.87 (12.3)	Both	S:19.93 (2.07) NS:21.12 (3.94)	S:6.07(0.86) NS:8.27(1.60)	AWGS 2019	(1) SMI: < 7 kg/m2 in male,< 5.7 kg/m2 in female, (2) grip strength: < 28.0 kg in male, < 18.0 kg in female, and (3) 6 m gait speed: walking speed <1 m/s	16 s rRNA sequencing of V3-V4,for alpha diversity and relative abundance
United States	Yuzefpolskaya et al. (36)	Hospitalized	264	ND	Both	ND	ND	Serum creatinine/cystatin-C (S-index)	ND	16 s rRNA sequencing for alpha diversity and relative abundance

S, Sarcopenia; NS, Non-Sarcopenia; Both, male and female; ND, No date; ASMI, Appendicular Skeletal; Muscle Index (kg/m^2^)^2^; SMI, Skeletal muscle index; BMI, Body mass index; ASMI, appendicular skeletal muscle mass index; aLM, low appendicular muscle mass; Mean, Arithmetical Mean; SD, ± standard; 16S rRNA, 16S Ribosomal Ribonucleic Acid; V4/V3–V4, regions of the 16S rRNA gene; S, Sarcopenic; NS, Not sarcopenic; HS, Handgrip strength; GS, Gait speed; 5XSST, Five Times Sit to Stand Test; 5CST, Five-times chair stand test; 6MWT, 6-min walk test; EWGSOP2, European Working Group on Sarcopenia in Older People 2; FNIH, Foundation for the National Institutes of Health sarcopenia project; ECS, European Consensus on Sarcopenia; AWGSG, Asian Working Group for Sarcopenia 2019 Guidelines; BMI, Body mass index; SPPB, Short-Physical Performance Battery; AWGS 2019, The Asian Working Group for Sarcopenia 2019; IWGS, International Working Group on Sarcopenia.

### 3.3 Quality assessment

We evaluated the 17 studies included in the meta-analysis on the NOS scale. One study scored 6 points, indicating low quality; 3 items scored 7 points, 8 items scored 8 points. For medium quality articles, 5 items scored 9 points, which was of high quality. Refer to Table 3 for detailed scoring rules.

**Table 3:** A maximum of two stars can be allotted in this category, one for gender, and one for another controlled factor.

Quality assessment of 17 studies on the newcastle-ottawa scale									
	Selection				Comparability control for important factor	Exposure			
Study	Adequatede finition of cases	Representativeness of the cases	Selection of controls	Definition of controls		Ascertainment of exposure	Same method of ascerainment for cases and controls	Nonresponse rate	Score
Picca et al. (31)	★	★	★	★	☆★	★	★	★	8
Yamamoto et al. (20)	★	★	★	★	★★	★	★	★	9
Ticinesi et al. (32)	★	★	☆	★	★★	★	★	★	8
Han et al. (26)	★	★	★	★	★★	★	★	★	9
Lee et al. (28)	★	★	★	★	★★	★	☆	★	8
Cox et al. (24)	★	★	★	★	☆★	★	★	★	8
Davis et al. (25)	★	★	☆	★	★★	★	☆	★	7
Ishida et al. (27)	★	★	☆	★	☆★	★	★	★	7
Kang et al. (17)	★	★	★	★	★★	★	★	★	9
Lee et al. (28)	★	★	★	★	☆★	★	★	★	8
Peng et al. (30)	★	★	★	★	★★	★	☆	★	8
Park et al. (29)	★	★	★	★	☆★	★	★	★	8
Wu et al. (13)	★	★	☆	★	★★	★	☆	★	7
Wang et al. (34)	★	★	★	★	★★	★	★	★	9
Yan et al. (35)	★	★	★	★	★★	★	★	★	9
Zhou et al. (18)	★	★	☆	★	☆☆	★	★	★	6
Yuzefpolskaya et al. (36)	★	★	★	★	★★	★	☆	★	8

### 3.4 Primary outcomes

#### 3.4.1 Alpha diversity

**Figure 2:**
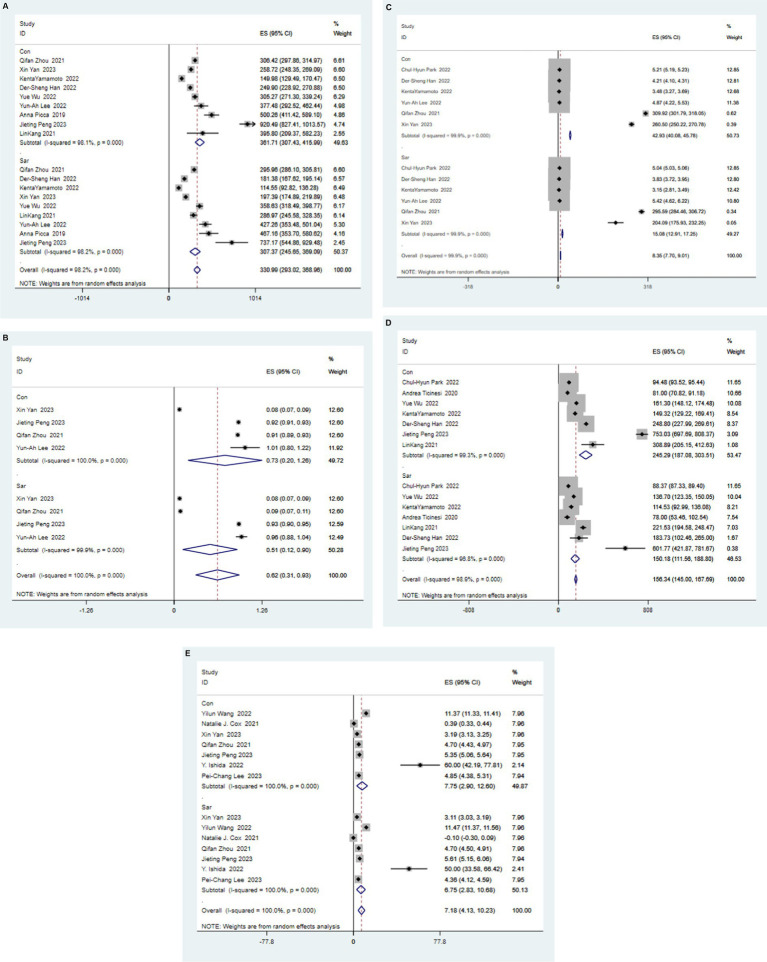
Forest map of alpha diversity differences by Chao index **(A)**, Simpson **(B)**, ACE **(C)**, Observed **(D)**, and Shannon Index **(E)**. WMD: weight mean difference; CI: confidence interval.

Alpha diversity reflect the diversity and abundance of microbes in a sample. Of the 17 articles included, 14 examined differences in alpha diversity between individuals with and without sarcopenia, of which 9 showed differences in alpha diversity between individuals with sarcopenia and those without sarcopenia, and 5 showed no differences (Table 4). Among these studies, 1 analyzed the V4 region, 11 analyzed the V3-V4 region, and 5 did not specify the region analyzed. After the meta-analysis, less muscle disease was observed in patients with the five types of gut microbe alpha diversity measure (Chao 1, Simpson, ACE, Observed, Shannon, Index) was relatively smaller than muscle disease populations, which dropped significantly, namely Chao1 [SAR: 307.37 (245 65, 369.09); N - SAR: 361.71 (307.43, 415.99)], Simpson [SAR: 0.51 (0.12, 0.90); N - SAR: 0.73 (0.20, 1.26)], the ACE [SAR: 15.08 (12.91, 17.25); N - SAR: 42.93 (40.08, 45.780)], observed [SAR: 150.18 (111.56, 188.80); N-SAR: 245.29 (187.08, 303.51)], Shannon [SAR: 6.75 (2.83, 10.68); N-SAR: 7.75 (2.90, 12.60)] (Table 5; Figure 2).

**Table 4:** Detailed Alpha diversity differences between Sar and N-Sar populations in included studies.

Article title	Observed	Chao1	ACE	Shannon	Simpson
Sarcopenia-related gut microbial changes are associated with the risk of complications in people with cirrhosis				NS	
Sarcopenia in community-dwelling older adults is associated with the diversity and composition of the gut microbiota		NS		NS	NS
Differences in the gut microbiome and reduced fecal butyrate in elders with low skeletal muscle mass		D		D	
Patients with low muscle mass have characteristic microbiome with low potential for amino acid synthesis in chronic liver disease		D		NS	
Gut Microbial, Inflammatory and Metabolic Signatures in Older People with Physical Frailty and Sarcopenia: Results from the BIOSPHERE Study		NS			
Characteristics of the fecal microbiome and metabolome in older patients with heart failure and sarcopenia	D	D		D	D
Sex-specific associations between gut microbiota and skeletal muscle mass in a population-based study	D				
Alterations in intestinal microbiota diversity, composition, and function in patients with sarcopenia	D	D			
The associations of butyrate-producing bacteria of the gut microbiome with diet quality and muscle health	D			D	
The composition of the gut microbiome differs among community dwelling older people with good and poor appetite				D	
Characterization of the gut microbiota in hemodialysis patients with sarcopenia		D	D	D	D
Association of Sarcopenia and Gut Microbiome in HF, LVAD and Heart Transplant				D	
Relationships between sarcopenia, nutrient intake, and gut microbiota in Chinese community-dwelling older women		D	D		
Population-based metagenomics analysis reveals altered gut microbiome in sarcopenia: data from the Xiangya Sarcopenia Study				NS	

D, Decline; NS, No significance; Blank, lack of original data in the included literature.

**Table 5:** Meta-analysis results of Alpha diversity differences between Sar and N-Sar populations.

Alpha-diversity	N-SAR	SAR	Result
Chao1	361.71 (307.43,415.99)	307.37 (245.65,369.09)	Decline
Simpson	0.73 (0.20,1.26)	0.51 (0.12,0.90)	Decline
ACE	42.93 (40.08,45.780)	15.08 (12.91,17.25)	Decline
Observed	245.29 (187.08,303.51)	150.18 (111.56,188.80)	Decline
Shannon	7.75 (2.90,12.60)	6.75 (2.83,10.68)	Decline

#### 3.4.2 Differences in the microbial composition

At the phyla level, the proportion of Actinobacteria and Fusobacteria decreased (Fusobacteria decreased by 28 to 20% and Actinobacteria decreased by 10 to 7%), whereas there was no significant change in other phyla. At the genus level, Akkermansia, Dialister, Dorea, f-Lachnospiraceae, f-Ruminococcaceae, g-*Coprococcus*, g-*Faecalibacterium*, g-*Megamonas*, g-*Phascolarctobacterium*, g-*Prevotella*, *Lachnoclostridium*, *Paraprevotella*, The proportion of Ruminococcus_2, *Streptococcus* and *Subdoligranulum* decreased, and f-Ruminococcaceae, G-*Faecalibacterium*, G-*prevotella*, *Lachnoclostridium* decreased significantly (downregulated 3%), whereas other bacteria decreased slightly (down 1%). *Alistipes*, *Bacteroidota*, *Escherichia*, *Eubacterium*_rectale_group, *Flavonifractor*, g-*Bacteroides*, g-*Lactobacillus*, o-Clostridiales. The proportion of g-Parabacteroides and *Shigella* increased, among which G-*Bacteroides* had the largest increase (upregulated 12%), the proportion of Parabacteroides and *Shigella* increased by 2%, and the other bacteria increased by 1%. (See Table 6).

**Table 6:** Differences in the microbial composition.

Genus	CON	SAR	Result	Percentage change
Akkermansia	0.01 (−0.00, 0.02)	0.00 (0.00, 0.00)	Decline	1% ~ 0%
Dialister	0.01 (−0.01, 0.02)	0.00 (−0.01, 0.01)	Decline	1% ~ 0%
Dorea	0.02 (−0.00, 0.04)	0.01 (−0.00, 0.02)	Decline	2% ~ 1%
f-Lachnospiraceae;g-	0.04 (−0.04, 0.12)	0.03 (−0.04, 0.10)	Decline	4% ~ 3%
f-Ruminococcaceae;g-	0.08 (0.01, 0.15)	0.05 (−0.00, 0.10)	Decline	8% ~ 5%
g-COPROCOCCUS	0.01 (−0.00, 0.01)	0.00 (−0.00, 0.00)	Decline	1% ~ 0%
g-Faecalibacterium	0.06 (0.03, 0.08)	0.03 (−0.00, 0.05)	Decline	6% ~ 3%
g-Megamonas	0.02 (0.00, 0.04)	0.01 (−0.00, 0.02)	Decline	2% ~ 1%
g-Phascolarctobacterium	0.03 (0.00, 0.07)	0.02 (−0.01, 0.05)	Decline	3% ~ 2%
g-Prevotella	0.09 (0.02, 0.16)	0.06 (0.01, 0.12)	Decline	9% ~ 6%
Lachnoclostridium	0.03 (−0.01, 0.07)	0.00 (0.00, 0.00)	Decline	3% ~ 0%
Paraprevotella	0.01 (0.01, 0.01)	0.00 (0.00, 0.00)	Decline	1% ~ 0%
Ruminococcus_2	0.02 (−0.00, 0.04)	0.01 (−0.01, 0.02)	Decline	2% ~ 1%
Streptococcus	0.03 (−0.01, 0.08)	0.02 (−0.02, 0.06)	Decline	3% ~ 2%
Subdoligranulum	0.02 (−0.00, 0.04)	0.01 (−0.00, 0.02)	Decline	2% ~ 1%
Alistipes	0.01 (−0.00,0.03)	0.02 (0.02, 0.02)	Rise	1% ~ 2%
Bacteroidota	0.02 (−0.02, 0.06)	0.03 (0.01, 0.04)	Rise	2% ~ 3%
Escherichia	0.01 (−0.02, 0.04)	0.02 (−0.04, 0.07)	Rise	1% ~ 2%
Eubacterium_rectale_group	0.01 (−0.02, 0.04)	0.02 (−0.04, 0.07)	Rise	1% ~ 2%
Flavonifractor	0.00 (−0.00, 0.01)	0.01 (0.00, 0.01)	Rise	0% ~ 1%
g-Bacteroides	0.03 (0.00, 0.07)	0.15 (0.03, 0.26)	Rise	3% ~ 15%
g-Lactobacillus	0.01 (−0.00, 0.03)	0.02 (−0.00, 0.04)	Rise	1% ~ 2%
o-Clostridiales;g-	0.02 (−0.01, 0.05)	0.03 (−0.01, 0.07)	Rise	2% ~ 3%
Parabacteroides	0.01 (0.00, 0.02)	0.03 (−0.02, 0.08)	Rise	1% ~ 3%
Shigella	0.03 (−0.02, 0.07)	0.05 (0.01, 0.09)	Rise	3% ~ 5%

### 3.5 Publication bias and sensitivity analysis

Alpha diversity and abundance of flora were evaluated by funnel figure inspection. The results showed that the left and right sides of the funnel were roughly symmetrical, indicating there was no obvious publication bias. We conducted a sensitivity analysis of the indicators with ≥3 articles and found that there was no study having large impact on the overall results, so the results can be considered stable. (The Figure is shown in the attachment).

## 4 Discussion

Changes in the gut microbiota have an important impact on the physiological processes, disease occurence and prognosis of many living organisms. The theory of the gut-muscle axis relates the number, type, and function of the gut microbiota to the function of skeletal muscle, and an increasing number of studies have focused on the role and mechanism of the gut microbiota in the pathogenesis of sarcopenia. For example, in a mouse model of chronic intestinal inflammation, restoration of commensal *E. coli* levels was effective in preventing skeletal muscle from atrophy (38). Therefore, the evaluation of the correlation between sarcopenia and gut microbiota has certain reference value for exploring the gut microbiota as a potential target for the regulation of sarcopenia treatment.

The current approach to treating sarcopenia mainly focuses on lifestyle changes, particularly physical exercise and nutritional support. Strength training exercises and increased protein intake are viewed as the primary treatments. Recent clinical trials have also investigated the potential of pharmacological interventions, including selective androgen receptor modulators (SARMs) and other emerging therapies (39, 40). However, no drug has yet been officially approved for the treatment of sarcopenia, making lifestyle modifications the most effective strategy available at present.

Measures of alpha diversity are widely used in the evaluation of samples to reflect the richness and diversity of the microbial community, and a higher alpha diversity is more beneficial to the host. The meta-analysis showed that the alpha diversity of the intestinal microbiome diversity in the sarcopenia population was significantly reduced compared to that in the non-sarcopenia population, suggesting that there were differences in the total number and richness of the intestinal microbiome in individuals with different muscle status, providing strong support for the role of the intestinal muscle axis in the course of sarcopenia.

The human gut microbiota is mainly composed of four phyla, namely the phylum of Farmicutes, Bacteroidetes, Proteobacteria, and Actinobacteria. The imbalance between these phyla can change the microenvironment in the gastrointestinal tract and then directly or indirectly lead to the occurrence of various diseases, such as obesity, allergy, type 1 and type 2 diabetes, and autism (41). At the phylum level, Firmicutes, Fusobacteria, Bacteroidetes, Proteobacteria, and Actinobacteria dominated the gut microbiota in our study samples. In particular, Actinobacteria and Fusobacteria were significantly reduced in people with sarcopenia compared to people without sarcopenia. Ruminococcaceae, Faecalibacterium, and Prevotella were the genera whose relative abundance was significantly reduced in the intestinal microbiota of patients with sarcopenia. Other studies have also shown that reduced abundance of intestinal flora is associated with decreased muscle mass and the development of other diseases. The relative abundance of Ruminococcaceae has been shown to be positively correlated with grip strength and can be used as a predictor of sarcopenia in patients with cirrhosis (17). Faecalibacterium abundance is widely regarded as one of the key components to promote health in the intestinal microbiota, and it is closely related to improving insulin sensitivity, anabolic balance, and reducing intestinal and systemic inflammation (42–44). However, Lin et al. (33) believed that the abundance of *P. praxis* was negatively correlated with skeletal muscle mass, which was contrary to our findings. This could be due to cross-feeding between bacteria. In fact, only in the presence of sufficient Bifidobacterium can Faecalibacterium play a role in the energy supply (45, 46). Lee et al. indicated that the quality of Prevotella through reduced muscle damage and function did not delay muscle disease (9). Additionally, decreased Prevotella levels is a well-known indicator of weakness, and P/B is considered as a biomarker that distinguishes control groups from individuals with sarcopenia (47). These results are consistent with the results of this meta-analysis. In contrast, Parabacteroides and Shigella are abundant in sarcopenia. Parabacteroides proved to be an increased bacterial genus in sarcopenia patients and could be used as a biomarker for the diagnosis of sarcopenia (48, 49). Shigella, as a bacterial pathogen, can increase skeletal muscle damage by triggering inflammasome activity and inducing an inflammatory response (50). One of the most important findings of this study is that Bacteroides is highly elevated in people with sarcopenia. As one of the most anaerobic bacteria, Bacteroides has an obvious negative correlation with age and can be used as an indicator to predict mortality in older individuals (16). In contrast, *Bacteroides plebeius* supplementation can counteract sarcopenia caused by chronic kidney disease through the Mystn/ActRIIB/SMAD2 pathway. The reason for the difference may be that supplementation with *Bacteroides plebeius* increases the content of Bacteroides, but does not increase the proportion of Bacteroides in the intestinal microbiota, which needs to be confirmed by more controlled experiments to explore the relative abundance of different bacteria.

### 4.1 Lifestyle changes in patients with sarcopenia affect the structure of the gut microbiota

Sarcopenia, as a degenerative disease with a high incidence in the older adults, is often associated with frailty and other chronic diseases, which jointly affect the motor system of patients and the function and stability of other systems, among which the gut microbiota is one of the targets. This may be due to a combination of factors such as changes in the diet structure of patients with sarcopenia, decreased physical activity, a history of surgery or anesthesia, and the taking of multiple medications for other diseases.

As aging, there is a natural tendency to reduce our food intake, which can lead to anorexia (51). This is attributed to the secretion and response disorders of some hormones that control food intake (such as cholecystokinin, etc.) (45), and is also related to factors such as social isolation, disease, and oral problems in the older aged population (52). Several studies have shown that dietary changes can cause permanent changes in intestinal flora, and this change in the dietary structure is reflected in the intake of various compounds (the three main energy substances). For example, different carbohydrate intake can result in different changes in the gut microbiome: complex carbohydrates can increase levels of beneficial bacteria, such as Bifidobacterium (53). Inulin and fructooligosaccharides in the oligosaccharides promote the growth of Bifidobacteria and Lactobacillus. Fructan promotes the growth of butyric-producing bacteria such as *Faecium prevotella* (54, 55). Vegetarians due to the intake of a large amount of cellulose, which results in a decrease in local gastrointestinal pH and a general decrease in Bacteroides (56). At the same time, it promotes the growth of Gram-positive bacteria that produce butyrate, enhances the barrier function of the gastrointestinal tract, and reduces the potential pro-inflammatory state (21, 57). In contrast, an unhealthy high-fat diet promotes taurine synthesis, which in turn stimulates the growth of pathogenic bacteria and induces low-grade intestinal inflammation. A recent meta-analysis showed that a high-fat diet increased the F/B ratio (58). Arumugam (59) also demonstrated that Bacteroides are highly correlated with saturated fat. Because skeletal muscle synthesis depends on protein intake, most older patients diagnosed with sarcopenia are advised to take additional protein supplements. However, studies have shown that a high-protein diet can stimulate the activity of enzymes such as β-glucosidase that produce toxic metabolites (60), improve the function of bile acids, and then cause damage to gastrointestinal mucosal function and changes in the microbiome. These findings support our determination of the relationship between dietary changes and structural changes in the intestinal microbiota in people with sarcopenia.

Sarcopenia patients are characterized by reduced muscle mass and strength, resulting in a greatly increased risk of falls and fractures. Due to the frequent occurrence of osteoporosis, most patients after falls and fractures require surgical treatment. Recent studies have shown that the stress process caused by orthopedic or other surgery can cause intestinal microbiota dysbiosis in rodents and humans, including reduction in intestinal microbial diversity and changes in composition (61). During surgery, anesthesia has the most obvious impact on the intestinal flora. The link between anesthetics and the gut microbiota can be explained by their affinity to neurotransmitters (62). Studies have shown that general anesthesia can interfere with intestinal lining metabolic stability by promoting gastrointestinal hemodynamic changes and reducing glucose absorption by the intestine (63–65). For example, the diversity of the fecal microbiota of mice 7 days after isoflurane anesthesia was significantly reduced, in which the richness of Firmicutes was decreased, and the richness of Proteobacteria and Actinobacteria increased. These changes in the microbiota are considered to be related to aging, which areharmful to the host (66).

Physical activity is emphasized in daily life as a recognized physical therapy that can effectively improve patients with sarcopenia. Recent studies have shown that exercise can serve as an effective way to reduce inflammation and can also produce positive effects on changing the composition of the gut microbiota (67, 68). However, people with sarcopenia often do not complete the expected intensity of exercise due to weakness or pain, which not only affects muscle strength recovery, but also affects the structure of the gut microbiome, which has been demonstrated in both humans and rodents. Allen (69) tested the fecal samples of people who underwent aerobic training and those that did not exercise for a period of 6 weeks using hue gas spectrometry and found that compared to the exercise group, the non-exercise group with low body mass index had significantly lower butyrate-producing bacteria, such as *Faecalibacter*. Recent studies in rodents have produced interesting results, with data suggesting that each type of exercise leads to changes in the gut microbiome (70). Wang (37) found that the abundance of *Prevotella*, *Ruminococcus*, and *Akkermansia* in inactive rats was significantly lower than in exercise-trained rats. At the same time, they also found that changes in the gut microbiota, such as the increase in *A. muciniphila*, were strictly dependent on exercise, with a significant decrease in the relative abundance of *A. muciniphila* after exercise training was withdrawn. Therefore, changes in the intestinal microbiota in sarcopenia may be determined by the combination of low physical activity and dietary changes caused by decreased energy expenditure in the gastrointestinal environment.

Older patients with sarcopenia inevitably take a variety of drugs, of which antibiotics can most affect the gut microbiota. As drugs commonly used in clinical practice, broad-spectrum antibiotics can eliminate or prevent bacterial colonization in the human body without targeting specific bacteria. As a result, broad-spectrum antibiotics can greatly affect the composition of the gut microbiota, reducing its diversity, and delaying colonization for a longer period of time after administration. Studies of Pallega have shown that a combination of *E. faecalis*, gentamicin and vancomycin can lead to strains of bifidobacteria and butyrate and increase rates of enterobacteriaceae and other pathogens (71). Ianiro (72) showed that the use of antibiotics reduced the microbial diversity. In addition to antibiotics, other drugs can also act on intestinal prokaryotes. A large-scale drug screening study reported that 27% of non-antibiotic drugs may cause growth arrest of a variety of gut bacteria (73). Recent studies also suggested that the gut microbiome may be critical for optimal muscle function (74). In fact, the use of antibiotics to deplete the microbiome can lead to decreased running ability and muscle contraction (75, 76).

The reason for the changes in the gut microbiota of patients with sarcopenia may also be related to the residential environment, ethnicity, and genetic factors of the sarcopenia population included. Skeletal muscle is an important part of the motor system, and its loss of strength or quality can directly or indirectly change the microbiota of the intestine, an immune organ, which in turn affect pathophysiological processes such as the systemic inflammatory response. This also provides us with a potential target for intervention in other systems in addition to the motor system to explore the treatment and prognosis of patients with sarcopenia.

### 4.2 Changes in the gut microbiota affect skeletal muscle status and function

The change in flora richness can indirectly influence muscle synthesis and breakdown and play a role in the pathogenesis of sarcopenia, which may link to the regulation of amino acid availability, the alteration of the ability to synthesize short-chain fatty acids, and the reduction of low-grade enteric inflammation. Studies in human and animal models have reported associations between the function of the gut microbiota involved in amino acid metabolism and individuals with sarcopenia. In this meta-analysis, the biosynthesis of phenylalanine tyrosine and tryptophan was reduced in the intestinal microbiome of participants with sarcopenia (77, 78). Phenylalanine and tyrosine can stimulate muscle protein synthesis and improve muscle strength. Tryptophan, an essential amino acid, is also believed to regulate skeletal muscle mass (79). Therefore, the reduction of the amino acid biosynthesis pathway may be a mechanism that leads to the pathogenesis of sarcopenia.

As a common age-related disease, sarcopenia is highly related to chronic inflammation in the body. Previous studies have shown that some strains can regulate metabolism by increasing the expression of antioxidant enzymes (80). *Lactobacillus plantarum*, *Lactococcus lactis*, and *Streptococcus thermophilus* can increase Superoxide Dismutase (SOD) activity (81). *Lactobacillus*, *Lactococcus*, and *Bifidobacterium* can increase intestinal glutathione (GSH) level and play an important role in removing hydroxyl free radicals (OH^−^) (81). In the regulation of muscle mass and function, the gut microbiome has been shown to influence skeletal muscle metabolism through chronic inflammation leading to synthetic muscle resistance. Abnormal microbial assemblages and age-related changes in intestinal mucosal permeability encourage various bacterial components, including lipopolysaccharide (LPS), to enter the bloodstream, thus increasing levels of inflammatory factors such as interleukin (IL)-6 and tumor necrosis factor (TNF)-α, thereby activating the inflammatory response (11). Conversely, gut microbes may also be involved in lowering inflammation levels by influencing metabolites. For example, increased abundance of *F. prausnitzii* can improve local intestinal and systemic inflammation (42–44), and Lactobacilli as a group of beneficial bacteria can improve the immune system, improve intestinal barrier function, and reduce the concentration of bacteria-derived endotoxins, LPS, and related inflammatory factors (82, 83).

SCFAs, one of the most well-known bacterial metabolites, can provide up to 10% of the daily energy needs of the body (84). Among them, butyrate, acetate, and propionate are the most well-known SCFAs, accounting for about 95% of the total. SCFAs plays an important role in regulating cell growth and differentiation. It has been shown to affect skeletal muscle mass by regulating epithelial cell function and protein synthesis pathways, increasing ATP production, promoting fat oxidation, and limiting muscle steatosis, reducing insulin sensitivity, and improving inflammation (85). Besten (86) demonstrated that SCFAs regulates skeletal muscle status by increasing the AMP/ATP ratio or activating AMPK through the FFAR2-leptin pathway. As the most functional short-chain fatty acid, butyrate plays an important role in the regulation of the immune system and the maintenance of intestinal barrier homeostasis in sarcopenia. Studies have shown that the butyrate produced by *Roseburia*, *Fusicantenibacter*, *Lachnoclostridium*, etc. has a significant effect on the function of skeletal muscle cells by promoting mitochondrial activity (87). At the same time, butyrate can also resist endotoxin translocation and reduce inflammation by enhancing the tight junction assembly and improving intestinal barrier function, down-regulating NF-κB and STAT2 activities (88, 89). In individuals without SCFAs, additional butyrate intake can affect energy metabolism in skeletal muscle tissue, thereby reversing muscle function. Walsh’s research proves that, added in 16-month-old mice, butyrate can inhibit histone beta deacetylase and have substantial protection against hind leg muscle atrophy (90).

In addition, the extra added probiotics are considered feasible nutritional intervention measures that can improve muscle mass and/or function and help prevent muscle-reducing disease. Studies have shown that oral probiotic supplements containing *Lactobacillus Roche* and *Lactobacillus galaei* reduce serum levels of pro-inflammatory cytokines and improve muscle mass (91). Another study also demonstrated that male athletes taking probiotics had improved muscle mass, strength, and exercise recovery (92).

In summary, the relationship between the intestinal microbiota and the pathophysiology of sarcopenia is complex and unclear, but our study can provide insights for future research. Diversity and relative richness may not directly explain the effects of the gut microbiota on muscle health, but the results of this study suggest that the application of GM as prevention and treatment of SAR has great potential, and the exploration of specific strains may be a worthwhile direction to improve SAR. Therefore, in the future, we can further study and analyze the results of all taxa to help remove confounding factors for the study of disease.

## 5 Study limitations

The present study selected the articles according to the inclusion and exclusion criteria of the study design, extracted data from the articles not reporting specific data by using software to precisely extract values from plot points, and operated strictly according to the project reporting guidelines of systematic review and meta-analysis. However, there are still some limitations: (1) The cross-sectional studies included in this study evaluated the composition of the intestinal microbiota rather than its function, so more cohort studies are needed to explore the causal relationship between GM and its metabolites and skeletal muscle mass and function. (2) There was heterogeneity between the results of the included studies. Similar to other observational studies, heterogeneity is an inevitable problem, which depends not only on the heterogeneity of clinical studies and statistical methods, but is also limited by the sample size, living place, dietary habits, physical exercise, drug use, and other factors of the participants, which tend to affect the composition of intestinal microorganisms. At the same time, researchers in different regions use different diagnostic criteria for sarcopenia when enrolling study samples, which also increases the heterogeneity between the association between skeletal muscle mass and strength and intestinal microbiota. (3) Although 16S rRNA analysis is a powerful technique, it cannot provide reliable in-depth identification, such as metagenomic sequencing, and sequencing platforms, regions, and methods vary from study to study, which does not guarantee that meta-analysis can evaluate all strains and their characteristics associated with sarcopenia. (4) The choice of different 16S rRNA gene regions, such as V3-V4 and V4, in microbiome studies can lead to variations in results due to differences in the resolution and coverage of microbial taxa. The V3-V4 region is often favored for its higher resolution and ability to distinguish closely related species. However, its longer length can introduce biases, affecting sequencing efficiency and accuracy (93, 94). Conversely, the V4 region, being shorter, is less prone to sequencing errors and is more compatible with high-throughput sequencing platforms, though it may offer lower taxonomic resolution (95, 96). Studies that fail to specify the 16S rRNA gene region used may lack consistency and comparability, potentially leading to discrepancies in the interpretation of microbiome data (97). To ensure the robustness of our findings, we used standardized bioinformatics pipelines, normalized data, and conducted sensitivity analyses. (5) This study identified specific changes in the microbiota associated with sarcopenia through the analysis of cross-sectional clinical studies. However, due to the absence of gene sequencing data on sarcopenia in public databases, we were unable to perform more detailed β-diversity and Mendelian randomization analyses. Furthermore, the data available from cross-sectional studies was insufficient to support comprehensive metabolic pathway analyses. These limitations hindered our ability to fully understand the causal relationship and metabolic functions between gut flora and sarcopenia. Future research should aim to obtain more gene sequencing data, incorporate diverse analytical methods, and explore the relationship between gut microbiota and sarcopenia in terms of metabolic pathways, to provide a more comprehensive insight.

## 6 Conclusion

In summary, this study observed differences in the composition of intestinal microbes between sarcopenic and non-sarcopenia populations at the phylum and genus levels. At the alpha diversity level, Chao, Shannon, ACE, Simpson, and the observed index in the sarcopenic group were reduced to varying degrees compared to those in the non-sarcopenia population. Meanwhile, in the included literature, the intestinal microbiota of sarcopenic patients was observed to decrease the abundance of f-Ruminococcaceae; g-*Faecalibacterium*, g-*Prevotella*, and *Lachnoclostridium* at the genus level, increase the abundance of g-*Bacteroides*, Parabacteroides, and *Shigella*, and decrease the proportion of *Actinobacteria* and *Fusobacteria* at the phylum level. However, heterogeneity exists in different studies and the relationship between the intestinal microbiota and sarcopenia is complex, involving a variety of pathophysiological processes and metabolic pathways, and the influence of many covariates, such as diet, region, polymorbidity, and polypharmacy, so the conclusion should be applied to other populations with caution. The internal relationship between them is not clear, and further research is needed to explore the role of microorganisms in muscle synthesis and decomposition, providing new ideas and methods for the prevention and treatment of sarcopenia exploiting the intestinal microbiota.

## Data availability statement

The original contributions presented in the study are included in the article/Supplementary material, further inquiries can be directed to the corresponding author.

## Author contributions

QS: Conceptualization, Data curation, Formal analysis, Funding acquisition, Investigation, Methodology, Project administration, Resources, Software, Supervision, Validation, Visualization, Writing – original draft, Writing – review & editing. YZ: Data curation, Investigation, Project administration, Resources, Software, Supervision, Validation, Visualization, Writing – review & editing. XiaL: Supervision, Writing – review & editing. HL: Supervision, Writing – review & editing. XZ: Supervision, Writing – review & editing. LX: Supervision, Writing – review & editing. SY: Supervision, Writing – review & editing. YW: Supervision, Writing – review & editing. XifL: Conceptualization, Data curation, Formal analysis, Investigation, Methodology, Project administration, Resources, Supervision, Validation, Writing – review & editing.
